# Supplementary material for: PSPC1 Potentiates IGF1R Expression to Augment Cell Adhesion and Motility
Source: Cells. 2020 Jun 18;9(6):1490. doi: 10.3390/cells9061490 (PMC7349238; doi:10.3390/cells9061490)
Supplement: Supplementary file 1 [file cells-09-01490-s001.pdf]

Supplementary information

PSPC1 potentiates IGF1R expression to augment cell adhesion and motility

Hsin-Wei Jen[1,2], De-Leung Gu [2], Yaw-Dong Lang [2] and Yuh-Shan Jou [1,2,*]

[1] Graduate Institute of Life Sciences, National Defense Medical Center, Taipei, Taiwan
[2] Institute of Biomedical Sciences, Academia Sinica, Taipei, Taiwan
* Author to whom correspondence should be addressed

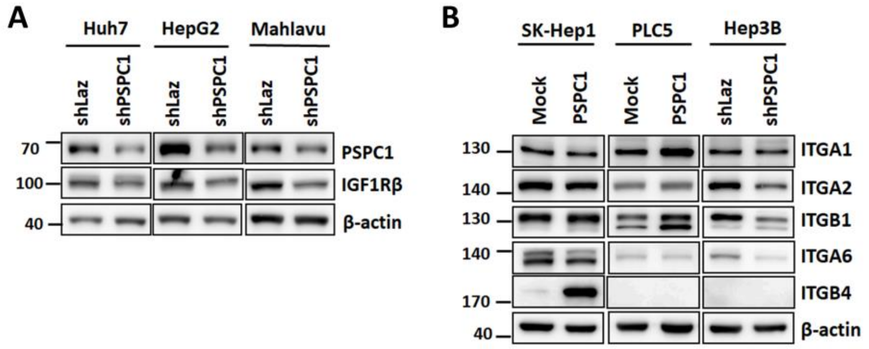

Supplementary Figure S1: Expression of IGF1R and integrin in PSPC1-expressing or PSPC1-depleted HCC cells by Western blotting analysis

(**A**) Detection of IGF1R protein levels in three PSPC1-knockdown cells Huh7, HepG2 and Mahlavu. (**B**) Detection of selected integrin expression in PSPC1-overexpressing or PSPC1-depleted HCC cells by using their total cell lysates immunoblotted with specific integrin antibodies as shown.

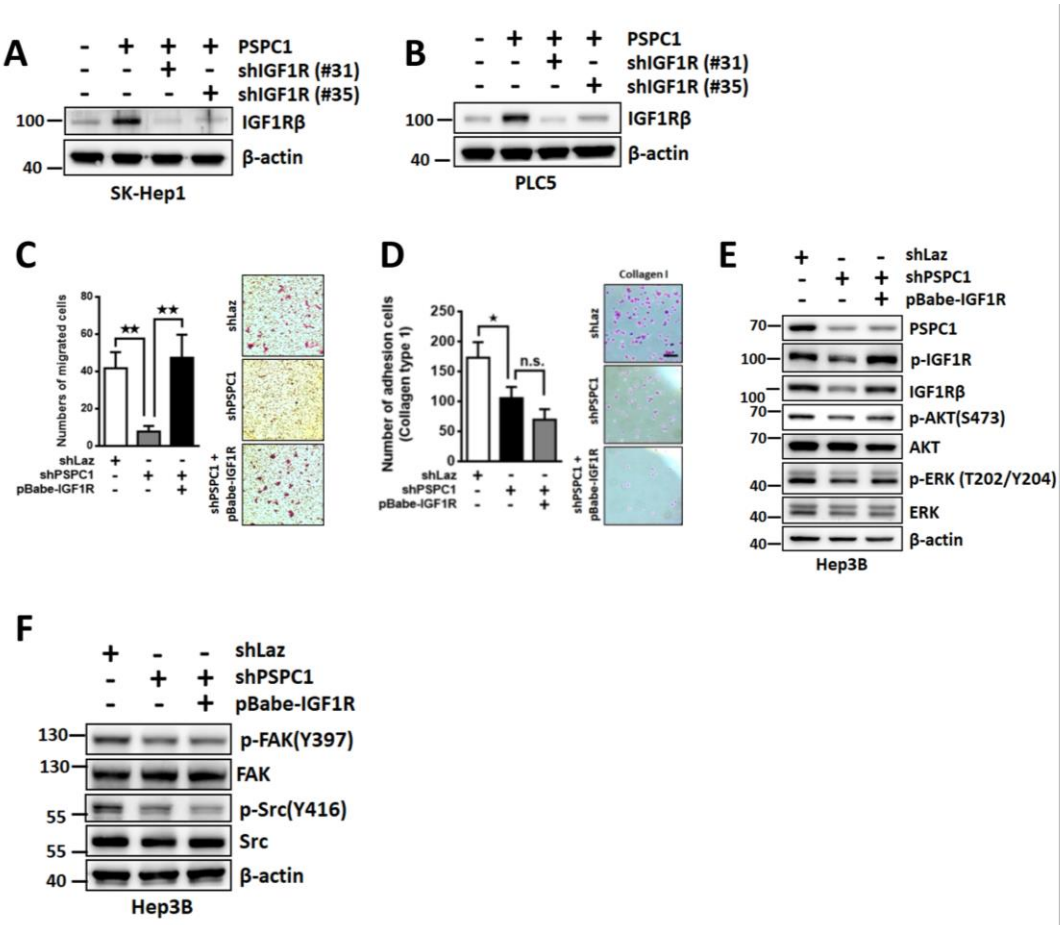

Supplementary Figure S2: PSPC1-modulated IGF1R downstream signaling in HCC cells.

(**A, B**) Immunoblotting of IGF1R expression in PSPC1-overexpressing SK-Hep1 and PLC5 cells treated with IGF1R shRNAs. (**C, D**) Cell migration and adhesion were measured in PSPC1-knockdown Hep3B cells rescued with exogenous expression of IGF1R. Exogenous expression of IGF1R in PSPC1-knockdown Hep3B cells were then applied for detection of altered AKT/ERK signaling including (**E**) total PSPC1, IGF1R, AKT, ERK, p-IGF1R, p-AKT(S473), and p-ERK(T202/Y204) as well as altered FAK/Src signaling including (**F**) total FAK, Src, p-FAK(Y397) and p-Src(Y416) by immunoblotting assay. Data are mean ± SD analyzed by paired and two-tailed *t*-test, n=3 per group, *p*-values (* $p < 0.05$; ** $p < 0.01$).

Supplementary Table S1

List of constructs

plasmid	Source
pcDNA3-HA PSPC1	Addgene (#101764)
pBABE-bleo IGF1R	Addgene (#11212)
pcDNA3-HA PSPC1 RRMmut	Homemade
pcDNA3-Flag PSPC1 ΔRRM	Homemade

List of shRNA and siRNA sequence

	Sequence
shPSPC1 #10	CCGGGCCTTGACTGTCAAGAACCTTCTCGAGAAGGTTCTTGACAGTCAAGGCTTTTTG
shPSPC1 #9	CCGGGAGCTGCTAGAGCAAGCATTTCTCGAGAAATGCTTGCTCTAGCAGCTCTTTTTG
shIGF1R #31	CCGGGAGACAGAGTACCCTTTCTTTCTCGAGAAAGAAAGGGTACTCTGTCTCTTTTTG
shIGF1R #35	CCGGCATGTACTGCATCCCTTGTGACTCGAGTCACAAGGGATGCAGTACATGTTTTTG
siFUS-1	CGGACAUGGCCUCAAACGAdTdT
siFUS-2	*ACAGCCCAUGAUUAAUUUGUAdTdT*
siNONO-1	GGGGUGGUAUUAAACAAGUCAdTdT
siNONO-2	GGAACAGGGUUACUGUAUACUdTdT
si*NEAT1*-1	GGAGGGCUAAUCUUCAACUdTdT
si*NEAT1*-2	AGUUGAAGAUUAGCCCUCCdTdT

List of primers for qRT-PCR

Gene name	Sequence
COL1A2	GAGGGCAACAGCAGGTTCACTTA
	TCAGCACCACCGATGTCCAA
COL5A2	CCAGGAGTTCCAGGTTTCAA
	CAACTGTTCCTGGGTCACCT
ITGA10	TCTTGGAGGTGGTTCAGACC
	AAAGAAGCCAAGCTTCCACA

GAPDH	AAGGCTGTGGGCAAGG
	TGGAGGAGTGGGTGTCG
PDGFRB	CAGCTCCGTCCTCTATACTGC
	GGCTGTCACAGGAGATGGTT
LAMA5	ACCCAAGGACCCACCTGTAG
	TCATGTGTGCGTAGCCTCTC
LAMB1	TGGCTGGTTACTATGGCGAC
	GCACAGTCGTCACATCTGGA
IGF1R	AAAAACCTTCGCCTCATCC
	TGGTTGTCGAGGACGTAGAA

List of 1st antibodies

Antibody	Source	Catalog Number
PSPC1 (G7)	SANTA CRUZ	sc-374387
AKT	Cell signaling	#9272
p-AKT	Cell signaling	#4060
ERK	Cell signaling	#4095
p-ERK	Cell signaling	#9101
p-Paxillin (Y118)	Cell signaling	#2541
Talin1/2	Abcam	ab11188
ITGB1	BD Biosciences	610467
ITGB4	BD Biosciences	611232
ITGA1	Abcam	Ab181434
ITGA2	BD Biosciences	611016
ITGA6	Cell signaling	#3750
β-actin	ProteinTech	600008-1-Ig
p-FAK (Y397)	Cell signaling	#3283
p-FAK(Y576/577)	Cell signaling	#3281
FAK	Cell signaling	#3285
p-Src (Y416)	Cell signaling	#2101
Src	Cell signaling	#2109
p-IGF1R (Y1135/1136)	Cell signaling	#3024
IGF1R	Cell signaling	#3027
Alexa Fluor 568 Phalloidin	Life Technologies	A12380
FLAG-tag M2	Sigma-Aldrich	F1804
DAPI	Sigma-Aldrich	D8417
p54(nrb)/NONO	SANTA CRUZ	G-1
FUS	Abcam	Ab23439

Supplementary Table S2: PSPC1-pulldown proteins detected by pulled down, sliced bands after SDS-PAGE separation and LC mass spectroscopy analysis

*OS= Organism Name; GN=Gene Name; PE=Protein Existence; and SV=Sequence Version

Gene symbol	Protein name*
PSPC1	Paraspeckle component 1 OS=Homo sapiens GN=PSPC1 PE=1 SV=1
HSP71	Heat shock 70 kDa protein 1A/1B OS=Homo sapiens GN=HSPA1A PE=1 SV=5
HSP7C	Heat shock cognate 71 kDa protein OS=Homo sapiens GN=HSPA8 PE=1 SV=1
XRCC6	X-ray repair cross-complementing protein 6 OS=Homo sapiens GN=XRCC6 PE=1 SV=2
MYH9	Myosin-9 OS=Homo sapiens GN=MYH9 PE=1 SV=4
K2C1	Keratin, type II cytoskeletal 1 OS=Homo sapiens GN=KRT1 PE=1 SV=6
ACTB	Actin, cytoplasmic 1 OS=Homo sapiens GN=ACTB PE=1 SV=1
MYH10	Myosin-10 OS=Homo sapiens GN=MYH10 PE=1 SV=3
FUS	RNA-binding protein FUS OS=Homo sapiens GN=FUS PE=1 SV=1
NONO	Non-POU domain-containing octamer-binding protein OS=Homo sapiens GN=NONO PE=1 SV=4
DDX5	Probable ATP-dependent RNA helicase DDX5 OS=Homo sapiens GN=DDX5 PE=1 SV=1
K1C9	Keratin, type I cytoskeletal 9 OS=Homo sapiens GN=KRT9 PE=1 SV=3
GRP75	Stress-70 protein, mitochondrial OS=Homo sapiens GN=HSPA9 PE=1 SV=2
DDX17	Probable ATP-dependent RNA helicase DDX17 OS=Homo sapiens GN=DDX17 PE=1 SV=2
SFPQ	Splicing factor, proline- and glutamine-rich OS=Homo sapiens GN=SFPQ PE=1 SV=2
CMC2	Calcium-binding mitochondrial carrier protein Aralar2 OS=Homo sapiens GN=SLC25A13 PE=1 SV=2
K22E	Keratin, type II cytoskeletal 2 epidermal OS=Homo sapiens GN=KRT2 PE=1 SV=2
LMNB1	Lamin-B1 OS=Homo sapiens GN=LMNB1 PE=1 SV=2
HNRPU	Heterogeneous nuclear ribonucleoprotein U OS=Homo sapiens GN=HNRNPU PE=1 SV=6
HNRPM	Heterogeneous nuclear ribonucleoprotein M OS=Homo sapiens GN=HNRNPM PE=1 SV=3
TBA1C	Tubulin alpha-1C chain OS=Homo sapiens GN=TUBA1C PE=1 SV=1
TBA1B	Tubulin alpha-1B chain OS=Homo sapiens GN=TUBA1B PE=1 SV=1
DREB	Drebrin OS=Homo sapiens GN=DBN1 PE=1 SV=4
MYH14	Myosin-14 OS=Homo sapiens GN=MYH14 PE=1 SV=2

ACTA	Actin, aortic smooth muscle OS=Homo sapiens GN=ACTA2 PE=1 SV=1
IF2B1	Insulin-like growth factor 2 mRNA-binding protein 1 OS=Homo sapiens GN=IGF2BP1 PE=1 SV=2
K1C10	Keratin, type I cytoskeletal 10 OS=Homo sapiens GN=KRT10 PE=1 SV=6
NOP56	Nucleolar protein 56 OS=Homo sapiens GN=NOP56 PE=1 SV=4
RPN1	Dolichyl-diphosphooligosaccharide--protein glycosyltransferase subunit 1 OS=Homo sapiens GN=RPN1 PE=1 SV=1
SYRC	Arginine--tRNA ligase, cytoplasmic OS=Homo sapiens GN=RARS PE=1 SV=2
ANM5	Protein arginine N-methyltransferase 5 OS=Homo sapiens GN=PRMT5 PE=1 SV=4
IF2B3	Insulin-like growth factor 2 mRNA-binding protein 3 OS=Homo sapiens GN=IGF2BP3 PE=1 SV=2
RFA1	Replication protein A 70 kDa DNA-binding subunit OS=Homo sapiens GN=RPA1 PE=1 SV=2
TBB5	Tubulin beta chain OS=Homo sapiens GN=TUBB PE=1 SV=2
HNRPQ	Heterogeneous nuclear ribonucleoprotein Q OS=Homo sapiens GN=SYNCRIP PE=1 SV=2
XRCC5	X-ray repair cross-complementing protein 5 OS=Homo sapiens GN=XRCC5 PE=1 SV=3
LMNB2	Lamin-B2 OS=Homo sapiens GN=LMNB2 PE=1 SV=3
ABCD3	ATP-binding cassette sub-family D member 3 OS=Homo sapiens GN=ABCD3 PE=1 SV=1
PSMD3	26S proteasome non-ATPase regulatory subunit 3 OS=Homo sapiens GN=PSMD3 PE=1 SV=2
NXF1	Nuclear RNA export factor 1 OS=Homo sapiens GN=NXF1 PE=1 SV=1
AIFM1	Apoptosis-inducing factor 1, mitochondrial OS=Homo sapiens GN=AIFM1 PE=1 SV=1
EIF3D	Eukaryotic translation initiation factor 3 subunit D OS=Homo sapiens GN=EIF3D PE=1 SV=1
DDX3X	ATP-dependent RNA helicase DDX3X OS=Homo sapiens GN=DDX3X PE=1 SV=3
SCFD1	Sec1 family domain-containing protein 1 OS=Homo sapiens GN=SCFD1 PE=1 SV=4
NOP58	Nucleolar protein 58 OS=Homo sapiens GN=NOP58 PE=1 SV=1
HNRPL	Heterogeneous nuclear ribonucleoprotein L OS=Homo sapiens GN=HNRNPL PE=1 SV=2
ALBU	Serum albumin OS=Homo sapiens GN=ALB PE=1 SV=2

SYDM	Aspartate--tRNA ligase, mitochondrial OS=Homo sapiens GN=DARS2 PE=1 SV=1
GNL3	Guanine nucleotide-binding protein-like 3 OS=Homo sapiens GN=GNL3 PE=1 SV=2
TBB6	Tubulin beta-6 chain OS=Homo sapiens GN=TUBB6 PE=1 SV=1
ACTN4	Alpha-actinin-4 OS=Homo sapiens GN=ACTN4 PE=1 SV=2
KHDR1	KH domain-containing, RNA-binding, signal transduction-associated protein 1 OS=Homo sapiens GN=KHDRBS1 PE=1 SV=1
HORN	Hornerin OS=Homo sapiens GN=HRNR PE=1 SV=2
EWS	RNA-binding protein EWS OS=Homo sapiens GN=EWSR1 PE=1 SV=1
PIGS	GPI transamidase component PIG-S OS=Homo sapiens GN=PIGS PE=1 SV=3
ODP2	Dihydrolipoyllysine-residue acetyltransferase component of pyruvate dehydrogenase complex, mitochondrial OS=Homo sapiens GN=DLAT PE=1 SV=3
LTV1	Protein LTV1 homolog OS=Homo sapiens GN=LTV1 PE=1 SV=1
FXR1	Fragile X mental retardation syndrome-related protein 1 OS=Homo sapiens GN=FXR1 PE=1 SV=3
PLST	Plastin-3 OS=Homo sapiens GN=PLS3 PE=1 SV=4
TCPG	T-complex protein 1 subunit gamma OS=Homo sapiens GN=CCT3 PE=1 SV=4
LRC40	Leucine-rich repeat-containing protein 40 OS=Homo sapiens GN=LRRC40 PE=1 SV=1
E2AK2	Interferon-induced, double-stranded RNA-activated protein kinase OS=Homo sapiens GN=EIF2AK2 PE=1 SV=2
SENP3	Sentrin-specific protease 3 OS=Homo sapiens GN=SENP3 PE=1 SV=2
NUP85	Nuclear pore complex protein Nup85 OS=Homo sapiens GN=NUP85 PE=1 SV=1
ISK4	Serine protease inhibitor Kazal-type 4 OS=Homo sapiens GN=SPINK4 PE=2 SV=1
TRAP1	Heat shock protein 75 kDa, mitochondrial OS=Homo sapiens GN=TRAP1 PE=1 SV=3
COR1C	Coronin-1C OS=Homo sapiens GN=CORO1C PE=1 SV=1
DDX52	Probable ATP-dependent RNA helicase DDX52 OS=Homo sapiens GN=DDX52 PE=1 SV=3
MYO6	Unconventional myosin-VI OS=Homo sapiens GN=MYO6 PE=1 SV=4
VATA	V-type proton ATPase catalytic subunit A OS=Homo sapiens GN=ATP6V1A PE=1 SV=2
EF2	Elongation factor 2 OS=Homo sapiens GN=EEF2 PE=1 SV=4
GUF1	Translation factor GUF1, mitochondrial OS=Homo sapiens GN=GUF1 PE=1 SV=1

TKT	Transketolase OS=Homo sapiens GN=TKT PE=1 SV=3
TRY1	Trypsin-1 OS=Homo sapiens GN=PRSS1 PE=1 SV=1
BCLF1	Bcl-2-associated transcription factor 1 OS=Homo sapiens GN=BCLAF1 PE=1 SV=2
ANM3	Protein arginine N-methyltransferase 3 OS=Homo sapiens GN=PRMT3 PE=1 SV=3
GUAA	GMP synthase [glutamine-hydrolyzing] OS=Homo sapiens GN=GMPS PE=1 SV=1
G3PT	Glyceraldehyde-3-phosphate dehydrogenase, testis-specific OS=Homo sapiens GN=GAPDHS PE=1 SV=2
EIF3L	Eukaryotic translation initiation factor 3 subunit L OS=Homo sapiens GN=EIF3L PE=1 SV=1
YBOX1	Nuclease-sensitive element-binding protein 1 OS=Homo sapiens GN=YBX1 PE=1 SV=3
G3BP1	Ras GTPase-activating protein-binding protein 1 OS=Homo sapiens GN=G3BP1 PE=1 SV=1
SYFB	Phenylalanine--tRNA ligase beta subunit OS=Homo sapiens GN=FARSB PE=1 SV=3
MYO1D	Unconventional myosin-Id OS=Homo sapiens GN=MYO1D PE=1 SV=2
PABP1	Polyadenylate-binding protein 1 OS=Homo sapiens GN=PABPC1 PE=1 SV=2
TCPA	T-complex protein 1 subunit alpha OS=Homo sapiens GN=TCP1 PE=1 SV=1
K1C14	Keratin, type I cytoskeletal 14 OS=Homo sapiens GN=KRT14 PE=1 SV=4
TFCP2	Alpha-globin transcription factor CP2 OS=Homo sapiens GN=TFCP2 PE=1 SV=2
NFL	eurofilament light polypeptide OS=Homo sapiens GN=NEFL PE=1 SV=3
NUCL	Nucleolin OS=Homo sapiens GN=NCL PE=1 SV=3
FETUA	Alpha-2-HS-glycoprotein OS=Homo sapiens GN=AHSG PE=1 SV=1
RBM14	RNA-binding protein 14 OS=Homo sapiens GN=RBM14 PE=1 SV=2
RS27A	Ubiquitin-40S ribosomal protein S27a OS=Homo sapiens GN=RPS27A PE=1 SV=2
PROX2	Prospero homeobox protein 2 OS=Homo sapiens GN=PROX2 PE=2 SV=3
RBM39	RNA-binding protein 39 OS=Homo sapiens GN=RBM39 PE=1 SV=2
H1T	Histone H1t OS=Homo sapiens GN=HIST1H1T PE=2 SV=4
HNRPK	Heterogeneous nuclear ribonucleoprotein K OS=Homo sapiens GN=HNRNPK PE=1 SV=1

ADT1	ADP/ATP translocase 1 OS=Homo sapiens GN=SLC25A4 PE=1 SV=4
VIME	Vimentin OS=Homo sapiens GN=VIM PE=1 SV=4
ADT2	ADP/ATP translocase 2 OS=Homo sapiens GN=SLC25A5 PE=1 SV=7
IPO5	Importin-5 OS=Homo sapiens GN=IPO5 PE=1 SV=4
CCD91	Coiled-coil domain-containing protein 91 OS=Homo sapiens GN=CCDC91 PE=1 SV=2
TOIP2	Torsin-1A-interacting protein 2 OS=Homo sapiens GN=TOR1AIP2 PE=1 SV=1
NUP62	Nuclear pore glycoprotein p62 OS=Homo sapiens GN=NUP62 PE=1 SV=3
SYLC	Leucine--tRNA ligase, cytoplasmic OS=Homo sapiens GN=LARS PE=1 SV=2
NMDE2	Glutamate receptor ionotropic, NMDA 2B OS=Homo sapiens GN=GRIN2B PE=1 SV=3
PLEC	Plectin OS=Homo sapiens GN=PLEC PE=1 SV=3
CHD7	Chromodomain-helicase-DNA-binding protein 7 OS=Homo sapiens GN=CHD7 PE=1 SV=3
PSMD7	26S proteasome non-ATPase regulatory subunit 7 OS=Homo sapiens GN=PSMD7 PE=1 SV=2
LPP	Lipoma-preferred partner OS=Homo sapiens GN=LPP PE=1 SV=1
MDHM	Malate dehydrogenase, mitochondrial OS=Homo sapiens GN=MDH2 PE=1 SV=3
AAAT	Neutral amino acid transporter B(0) OS=Homo sapiens GN=SLC1A5 PE=1 SV=2
SRP68	Signal recognition particle subunit SRP68 OS=Homo sapiens GN=SRP68 PE=1 SV=2
EFHC1	EF-hand domain-containing protein 1 OS=Homo sapiens GN=EFHC1 PE=1 SV=1
HV306	Ig heavy chain V-III region BUT OS=Homo sapiens PE=1 SV=1
SFI1	Protein SFI1 homolog OS=Homo sapiens GN=SFI1 PE=1 SV=2
ZN550	Zinc finger protein 550 OS=Homo sapiens GN=ZNF550 PE=2 SV=2
CCD25	Coiled-coil domain-containing protein 25 OS=Homo sapiens GN=CCDC25 PE=1 SV=2
RBSK	Ribokinase OS=Homo sapiens GN=RBKS PE=1 SV=1
PUF60	Poly(U)-binding-splicing factor PUF60 OS=Homo sapiens GN=PUF60 PE=1 SV=1
K1683	Uncharacterized protein KIAA1683 OS=Homo sapiens GN=KIAA1683 PE=2 SV=1
CWC22	Pre-mRNA-splicing factor CWC22 homolog OS=Homo sapiens GN=CWC22 PE=1 SV=3
TR150	Thyroid hormone receptor-associated protein 3 OS=Homo sapiens

	GN=THRAP3 PE=1 SV=2
MPEG1	Macrophage-expressed gene 1 protein OS=Homo sapiens GN=MPEG1 PE=2 SV=1
Z286A	Zinc finger protein 286A OS=Homo sapiens GN=ZNF286A PE=2 SV=1
MRFL	Myelin regulatory factor-like protein OS=Homo sapiens GN=MYRFL PE=2 SV=2
MYO1B	Unconventional myosin-Ib OS=Homo sapiens GN=MYO1B PE=1 SV=3
KPYM	Pyruvate kinase PKM OS=Homo sapiens GN=PKM PE=1 SV=4
ZN318	Zinc finger protein 318 OS=Homo sapiens GN=ZNF318 PE=1 SV=2
EPHA5	Ephrin type-A receptor 5 OS=Homo sapiens GN=EPHA5 PE=1 SV=3
T2FA	General transcription factor IIF subunit 1 OS=Homo sapiens GN=GTF2F1 PE=1 SV=2
AN36B	Ankyrin repeat domain-containing protein 36B OS=Homo sapiens GN=ANKRD36B PE=2 SV=4
CCD22	Coiled-coil domain-containing protein 22 OS=Homo sapiens GN=CCDC22 PE=1 SV=1
EF1A1	Elongation factor 1-alpha 1 OS=Homo sapiens GN=EEF1A1 PE=1 SV=1
CNO11	CCR4-NOT transcription complex subunit 11 OS=Homo sapiens GN=CNOT11 PE=1 SV=1
NOV	Protein NOV homolog OS=Homo sapiens GN=NOV PE=1 SV=1
HMMR	Hyaluronan mediated motility receptor OS=Homo sapiens GN=HMMR PE=1 SV=2
SYC2L	Synaptonemal complex protein 2-like OS=Homo sapiens GN=SYCP2L PE=1 SV=2
SETB2	Histone-lysine N-methyltransferase SETDB2 OS=Homo sapiens GN=SETDB2 PE=1 SV=2
SSF1	Suppressor of SWI4 1 homolog OS=Homo sapiens GN=PPAN PE=1 SV=1
RREB1	Ras-responsive element-binding protein 1 OS=Homo sapiens GN=RREB1 PE=1 SV=3
CFA44	Cilia- and flagella-associated protein 44 OS=Homo sapiens GN=CFAP44 PE=1 SV=1
